# The Malaysian consensus statement on utilisation of cardiac CT

**DOI:** 10.2349/biij.4.4.e41

**Published:** 2008-10-01

**Authors:** KH Sim, YF Abdul Aziz, SP Chin, FL Chong, GH Choo, D Chew, ELM Ho, H Chia, MR Yusoff, KH Ng, SM Syed Abu Bakar, KH Tan, Z Musa

**Affiliations:** 1 National Heart Association of Malaysia, Damansara Uptown, Petaling Jaya, Selangor, Malaysia; 2 College of Radiology Malaysia, Department of Biomedical Imaging, University Malaya Medical Centre, Kuala Lumpur, Malaysia

## INTRODUCTION

Cardiovascular disease is a conglomerate of diseases that affect the heart and the arterial system in the body. It remains the number one cause of death in developed countries. In Malaysia, between the years 2000 and 2004, 20-25% of deaths in Government hospitals were due to cardiovascular disease [1].

Coronary artery disease (CAD) is characterised by the presence of atherosclerotic plaques in the coronary arteries. Coronary artery calcification is part of the development of these atherosclerotic plaques. These plaques progressively narrow the arterial lumen and hence impair blood flow. The reduction in coronary artery flow may be asymptomatic or symptomatic, may occur with or without exertion, and may culminate in a myocardial infarction, depending on the severity of the obstruction and rapidity of development.

Various investigational modalities are available for the detection of CAD. These include electrocardiography, echocardiography and radionuclide imaging. Lately, there has been increasing awareness in newer imaging techniques such as Computer Tomography (CT) and Magnetic Resonance Imaging (MRI) in diagnosing CAD.

CT as an imaging modality has been around for more than three decades. However, only in the last few years with the introduction of the multislice computer tomography (MSCT), has it allowed adequate imaging and interpretation of the status of the coronary arteries and its related structures. It is a novel technique, in that it allows non-invasive visualisation of the coronary artery lumen. Although the gold standard for diagnosing obstructive coronary disease is still invasive coronary angiography, cardiac CT, in certain clinical situations, may be an acceptable alternative [2,3,4,5]. Coronary calcium, a marker of plaque burden, can also be quantified with this method, and to a certain extent, plaque composition can be characterised [6].

The growth and availability of MSCT services in Malaysia have been immense. Therefore, it is important for the local medical profession to understand the requirements to obtain a minimum dataset for adequate interpretation of the cardiac study, and most importantly patient selection, preparation and safety. In order to make full use of this powerful imaging tool in daily clinical practice to increase the diagnostic yield, its potential and limitations have to be understood. This document aims to outline the requirements for cardiac CT, current indications, safety, reporting and training issues in Malaysia. The committee understands that CT technology and applications are evolving rapidly, and it is only pertinent that this document will be constantly revised to parallel the developments.

## RATIONALE OF CARDIAC CT

Comprehensive cardiac assessment requires information on coronary vascular anatomy, cardiac morphology, function, perfusion, metabolism and tissue characterisation. At the moment, no single imaging modality is able to successfully achieve accurate global assessment of the heart, which is a difficult organ to image because of its rapid, complex, cyclical, variable rate-dependent motion and its small vessels.

Cross-sectional CT imaging of the coronary arteries was first performed with the electron beam CT in 1984. However, low spatial resolution and high image noise limited the image quality. Since 1998, with the introduction of the 4-slice mechanical CT scanners and subsequently 16- and 64-slice CT scanners with higher spatial and temporal resolutions, accurate visualisation of the coronary arteries, cardiac anatomy as well as functional imaging are possible. At the time of writing, the evolving CT technology has presented us with various new developments, including dual source CT scanners as well as 320-slice CT scanners (which boast greater anatomical coverage and shorter scan time).

Invasive imaging techniques, especially selective conventional coronary angiography, will remain vital in planning and guiding catheter-based and surgical treatment of significantly stenotic coronary lesions. However, because coronary angiography is associated with a small but not negligible risk of complications (inherent in invasive procedures), inconvenience to patients and significant costs, coronary CT angiography (CTA) is an attractive alternative to invasive selective coronary angiography, with the potential to reduce the number of purely diagnostic angiograms. In particular, patients with intermediate likelihood of CAD may benefit from coronary CTA [7].

Cardiac CT is usually performed as a two-part examination – first, the coronary artery calcium score, and secondly, the coronary artery CT angiogram.

The calcium score measures the amount of calcified plaques in the coronary arteries as a surrogate marker for atherosclerotic disease. The majority of published studies have reported that the total amount of coronary calcium (usually expressed as the Agatston score) predicts coronary disease events beyond standard risk factors. Calcium score is useful given a negative CT test as atherosclerotic disease is less likely (negative predictive value 96-100%). In addition, intermediate risk patients (10-20% 10-year Framingham Risk Score) may benefit from a calcium score by refining clinical risk prediction and selecting patients for more aggressive risk factor modification and pharmacological intervention [8, 9].

Coronary CTA has been shown to be accurate in the detection and quantification of haemodynamically significant stenosis. Several studies have shown that the overall sensitivity is between 95% and 99%, and specificity 93-96% for detection of coronary artery stenosis [10-12]. Sensitivity, specificity, and the negative predictive value (NPV) of 64-slice MSCT per patient are approximately 97%, 79%, and 96%, respectively [13]. In addition, coronary CTA is able to display non-calcified plaque and vascular remodelling with good correlation with intravascular ultrasound [7].

The dataset obtained during the contrast-enhanced coronary scans can also be processed to obtain functional information of the heart.

This document represents a joint effort between the National Heart Association of Malaysia and the College of Radiology (Malaysia) to:

1. Present a summary of existing medical literature and data on cardiac CT. As this is an emerging technology, there are still areas of uncertain significance which are being explored in current studies. The appropriate and optimal application of this technology must be individually tailored to each patient taking into account risks, benefits, cost effectiveness and availability of alternative technology.
2. Make suggestions regarding the training of physicians wishing to participate in this field. The current criteria are formulated based on prevailing practices and conditions in Malaysia with reference to accepted standard clinical practices worldwide.

## REQUIREMENTS OF CARDIAC CT

Imaging of the heart using CT demands exact performance requirements from the scanner. These requirements include:

### (a) Minimisation of cardiac motion artifacts

This is the most critical aspect of cardiac imaging. The coronary arteries go through a series of complex movements during the cardiac cycle. Therefore, in order to image the coronary artery successfully, a scanner with a high temporal resolution is needed. One method of achieving this is by reducing the gantry rotation time [14]. The 4-slice CT scanners have rotation times of 0.5 sec. Newer scanners have gantry rotation times of 0.42 sec or less. Currently a 64-slice CT scanner has a temporal resolution of 165 msec.

Furthermore, the artifacts from cardiac motion can be further suppressed by imaging during the quiescent phase of the cardiac cycle [14]. This can be achieved by synchronising the image acquisition and image reconstruction with the ECG signal of the patient. This is done by using cardiac gating mechanism during scanning. Two ECG gating techniques are employed namely prospective ECG triggering and retrospective ECG triggering.

Another method that can be employed to improve the temporal resolution is to combine projection data from consecutive cardiac cycles [14]. This is called multi-segment reconstruction and provides a significant improvement in the temporal resolution by combining data from as many as four cardiac cycles. However, this technique relies heavily on the regularity of the heart rate for its success [15].

### (b) Minimisation of respiratory artifacts

Cardiac CT scans are performed during a single breath-hold to minimise motion from respiration. In order to achieve this, the acquisition time for the total coverage of the heart has to be short. The acquisition time for cardiac scans using the 4-slice CT scanners ranged between 33 and 40 sec. With the introduction of 16-slice CT scanners, the acquisition time was reduced to less than 20 sec. Further reduction in acquisition time (of less than 10 sec) was achieved with the 64-slice CT scanners, further reducing the occurrence of involuntary motion artifacts from respiration and movements of the patients.

### (c) High spatial resolution

The epicardial coronary arteries are small with diameters ranging from 5 mm proximally to less than 1 mm distally. Imaging of these small structures requires high spatial resolution. With the introduction of the 16-slice CT scanners, the presence of submillimetre detector widths has provided significant improvement in the in-plane (x-y) resolution. The current 64-slice CT scanners have detector widths ranging between 0.5 and 0.625 mm [14].

### (d) Adequate and uniform contrast enhancement

Poor vessel opacification is one of the factors that affect the image quality of the CT examination and renders distal vessels to be non-evaluable. Therefore, contrast injection protocol must be tailored to optimise contrast-to-noise ratio and to obtain uniform contrast enhancement. This is achieved by using contrast delivery techniques such as the automatic bolus tracking or the test bolus technique. Contrast needs to be administered via a powered contrast medium injector.

### (e) Employment of radiation dose-reduction techniques

Managing radiation dose to the patient during a cardiac CT scan is a primary concern. Various methods for dose-reduction are available. During the scan, the tube current (mA) can be modulated to be optimum at time of the targeted phase acquisition (usually diastolic). At other times, the tube current is kept at a nominal level. Using this technique, a dose saving of about 45% can be achieved, depending on the heart rate during the acquisition [14].

### (f) Management of large volume of image data

Cardiac CT using submillimetre detector thickness results in a large amount of image data to be reconstructed and interpreted. This provides a challenge to the clinician in management of these data. CT scanners currently need to be equipped with software, which enable these data to be visualised in the native axial images, multiplanar reconstruction (MPR), maximum intensity projection (MIP) as well as 3D volume rendering images. In order to do this, powerful workstations are needed for image reconstructions and evaluation. Software for measurement including quantitative manual and semiautomatic tools is useful for further analysis of coronary artery stenosis [14].

In view of the above requirements, it is the recommendation of the committee that the acceptable imaging systems that can be utilised for cardiac imaging include a minimum of a 16-slice MSCT, electron-beam CT or the dual-source CT scanner. At the very minimum, a 4-slice CT scanner is required for calcium scoring.

## INDICATIONS OF CORONARY CTA

As coronary CTA is a relatively new imaging modality, there is limited clinical evidence available for many indications and case scenarios for which coronary CTA may be useful. The committee has proposed an ‘Appropriateness Criteria’ championed by the American College of Cardiology Foundation [16] which blends scientific evidence and practical experience by engaging a diverse technical panel to rate each indication as appropriate or inappropriate application of coronary CTA. In formulating a consensus, these authors are greatly aided by the report of the American College of Cardiology Foundation Quality Strategic Directions Committee Appropriateness Criteria Working Group that included the American College of Radiology, Society of Cardiovascular Computed Tomography, Society for Cardiovascular Magnetic Resonance, American Society of Nuclear Cardiology, North American Society for Cardiac Imaging, Society for Cardiovascular Angiography and Interventions, and Society of Interventional Radiology [17].

1. An **appropriate** imaging study is one in which the expected incremental information, combined with clinical judgment, exceed the expected negative consequences by a sufficiently wide margin for a specific indication that the procedure is generally considered acceptable care and a reasonable approach for that indication (negative consequences include the risks of radiation or contrast exposure and test inaccuracies).
2. An **inappropriate** test for that indication means that cardiac CTA is not generally acceptable and is not a reasonable approach for that indication.
3. In many instances, cardiac CTA may be regarded as generally acceptable or a reasonable approach for that indication but would require more research and/or patient information to classify that indication definitively. Examples of such indications are detection of CAD in patients with acute chest pain but without typical ECG or cardiac enzyme changes; patients with typical chest pain and previous bypass graft or coronary stenting procedure; and screening for CAD in the asymptomatic high-risk population; and evaluation of left ventricular function in clinical heart failure patients with technically limited images from echo.

In deliberating the appropriateness of cardiac CTA, the committee evaluated only clinical evidence from cardiac CTA imaging using slice collimations of less than 1.0 mm for the detection of haemodynamically significant coronary artery stenosis – usually accepted as 50% luminal stenosis or greater [8]. Any local data that is more relevant to the Malaysian population were also included [18, 19]. The indications are for coronary imaging and calcium scoring unless otherwise specified. Provided that image quality is adequate, evaluation is performed by cardiac CTA level 2 or 3 trained doctors (see under Section: Training), and the patients are properly chosen and prepared for the study. Cardiac CTA has been investigated and reported for the following indications:

- Detection of haemodynamically significant coronary artery disease in a heterogeneous group of patients.
- Coronary stent and bypass graft patency.

### Appropriate Indications

#### 1. Detection of CAD in patients with:

- Chest pain and intermediate pre-test probability of CAD [20] and:
  - not suitable for exercise treadmill test (ETT);
  - un-interpretable or equivocal functional tests (ETT, perfusion or stress echo);
  - no ischaemic ECG changes and negative serial enzymes.
- Asymptomatic and low-to-moderate cardiovascular risk [21] but positive stress ECG;
- Cardiomyopathy to exclude coronary disease.

#### 2. Structure and Function Evaluation

- Suspected coronary anomalies;
- Assessment of complex congenital heart disease;
- Evaluation of intra-cardiac masses and pericardial diseases in patients with
- technically limited images echo, MRI or TEE;
- Evaluation of pulmonary vein anatomy prior to radiofrequency ablation for atrial
- fibrillation;
- Coronary vein mapping prior to placement of biventricular pacemaker.

### Inappropriate Indications

#### 1. Detection of CAD in patients with:

- Typical cardiac chest pain and high pre-test probability;
- Acute chest pain, high pre-test probability demonstrating ischaemic ECG changes and/or positive cardiac enzymes;
- Evidence of moderate-to-severe ischaemic on functional tests (ETT, perfusion, stress echo).

#### 2. Asymptomatic patients/population with:

- Low-to-moderate CV risk score for screening;
- High CV risk but CCTA or conventional angiography was normal up to 2 years previously.

#### 3. Asymptomatic patients/population without inducible ischaemia on functional tests for:

- Evaluation of bypass grafts;
- Evaluation of coronary stents.

### Indications for Calcium score

Recent publications suggest calcium scoring provides additional prognostic information to traditional risk scoring methods. Calcium scoring is appropriate for patients deemed at **medium risk for cardiovascular events** based on risk factor models. The presence of calcium and the incremental levels of coronary artery calcium score denote higher levels of risk and hence, guide the aggressiveness of preventive therapies and target goals. This is because there are no well-defined boundaries of risk levels but instead a risk continuum relationship similar to hypertension. Calcium scoring has been shown to be independently predictive of cardiovascular risk and adds incremental prognostic information to the conventional risk factor scoring methods [22-24].

## REPORTING OF CARDIAC CTA

### Format

The documentation of a CTA report should include the following headings:

1. Patient demographics
2. Reporting physician(s)
3. Type of examination
4. Indication for CT angiography
5. CT hardware and acquisition protocols (scanning and contrast)
6. Need for beta-blocker or any adjunctive medications and dosage used
7. Adequacy of image quality
8. Contrast used and amount
9. Findings
10. Complication(s) if any
11. Conclusion(s)
12. Recommendations

### Reporting

The CTA volume dataset must be examined in multiple reconstruction protocols including axial, multiplanar, and maximum intensity projection (MIP) [25].

Axial images are the most valuable in evaluation of the coronary arteries [7, 26]. Advanced post-processing tools e.g. 3D-Volume-rendered techniques, MIP are most accurate when viewed together with the axial data.

Proper window width and level or appropriate kernel should be used to visualise structure of interest or excessive calcification, if present.

**Figure 1:**
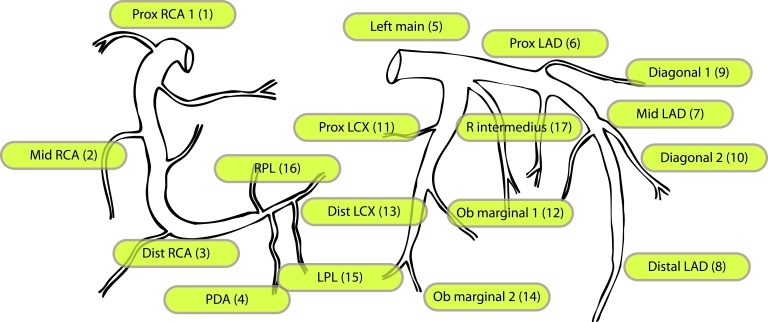
Modified 17-segment of the AHA reporting system [28].

Every segment of the coronary tree needs to be examined and documented using established nomenclature. This paper proposes one model of the coronary tree segmentation (Figure 1). This would allow cross-referencing with findings during catheter angiography. It also provides standardisation in the reporting which is crucial for clinical trials and communication. Presence of non-assessable segments should be noted.

In reporting the coronary arteries, dominance, presence of variants and anomalies should be noted. Description of plaques should include segmental location, extent of disease, attenuation characteristics, degree of stenosis and presence of vessel remodelling.

Stenosis quantification is based on referencing to adjacent segment’s luminal calibre rather than vessel luminal wall to outer wall diameter. Vessel cross-sectional and longitudinal views should be used for estimation [25]. Stenosis quantification can be performed by visual estimate and/or electronic callipers. Lesion severity are usually categorised as mild, moderate, severe or occluded which corresponds to stenotic severities of <50%, 50-70%, >70% and 100% narrowing. Plaque attenuation characteristics may be described e.g. calcified, non-calcified or mixed. Special features e.g. plaque ulceration, vessel dissection, and thrombus may sometimes be seen.

Additional information may be reported by experts if optimal images are available e.g. cardiac chambers (size, morphology), myocardium (thinning, contour change or attenuation difference), pericardium (masses, thickness, calcification, effusion) and extra-cardiac structures.

Information on cardiac function and valve structures, and function may be obtained with other post-processing methods if needed.

### Bypass graft assessment

Volume-rendering technique (VRT) displays good 3D-orientation of the grafts and the target anastomostic sites [2, 27]. Venous grafts are well visualised by CTA owing to its larger size and lack of mobility. Graft disease and stenosis are reported in a manner similar to native coronary arteries. Presence of surgical clips and often dense calcification in native vessels of post-CABG patients may give rise to artifacts that make assessment rather challenging.

### Stent assessment

Limited reports are currently available on stent evaluation with MSCT [27]. The accuracy of assessment is influenced by the type and size of stents. Owing to the high attenuation from stent material, appropriate window width and level or kernels should be chosen. Intrastent filling defects owing to in-stent restenosis (ISR) / occlusion should be noted. Adequacy of stent expansion may explain the reason for restenosis.

## RADIATION PROTECTION IN CARDIAC CT

The major drawback of imaging of the heart using multislice MSCT is the high radiation dose. The effective dose for coronary angiography using EBCT ranges from 1.5 to 2.0 mSv while a similar examination using 4-slice MSCT ranges from 6 to 13 mSv [29]. Recent MSCT multicentre studies have documented higher radiation doses of up to 30 mSv in day-to-day practice. However, with dose reduction techniques such as ECG-dependent dose modulation as mentioned before, dose saving of about 45% can be achieved. In a study by Hausleiter et al., the use of ECG-dependent dose modulator resulted in a significant reduction in the effective dose estimate from 10.6 ± 1.2 to 6.4 ± 0.9 (using 16-slice MSCT scanner) and 14.8 ± 1.8 to 9.4 ± 1.0 mSv (using 64-slice MSCT scanner) [30]. The new ‘step and shoot’ technique available in some systems today allows a 50-80% dose reduction [31].

It is imperative then that the principles of radiation exposure namely justification, limitation and ALARA (As Low As Reasonably Achievable) should be upheld at all times. Therefore, in view of the considerable radiation doses, it is the recommendation of the committee that:

1. The decision to expose a patient to cardiac CT has to be made by physicians aware of the radiation risks that will be incurred by the patient. At the time of writing, CT coronary artery angiography is not considered as a screening procedure and each patient should have justifiable indications to undergo the procedure. The use of MSCT for calcium scoring for risk stratification purposes are acceptable in clinical practice.
2. The use of dose-reduction techniques should be employed whenever possible. Effective dose during cardiac CT should ideally not exceed 13 mSv [29]. Special care should be taken when imaging children and young female (as the breast would be included in the scanning field).

## SAFETY ISSUES IN CARDIAC CT

Safety issues in cardiac CT involve issues regarding radiation, intravenous contrast administration and administration of β-blockers and nitrates.

Issues regarding radiation have been mentioned before. It is important that all personnel involved in cardiac CT are aware of radiation risks and undergo basic training in radiation effects and radiation protection.

Risks with contrast media administration include adverse reactions to the contrast, its nephrotoxic properties and risk of extravasation. The supervising physician should be able to identify patients with increased risk of developing adverse reactions or in which contrast media is contraindicated. Patients with renal failure should also be identified. The physician should be well versed in treating and managing contrast media reactions.

β-blockers are currently the preferred method to reduce heart rate. Supervising physicians should be aware of the dosage, action and the contraindications of β-blockers.

## TRAINING IN CARDIAC CT

CT is an important imaging modality for the detection and characterisation of cardiac disease; therefore it is crucial that physicians who supervise and interpret cardiac CT should have appropriate competency, experience and expertise [32].

Competencies are divided into:

1. Criteria for performing calcium scoring exclusively only.
2. Criteria for interpreting cardiac CT
  - level 1 competency
  - level 2 competency
  - level 3 competency

### 1. Criteria for performing calcium scoring exclusively only

Any physicians such as cardiologists and radiologists who are certified from the acceptable body/board and have undergone training which includes cardiac anatomy and experience with training in interpretation of cross-sectional imaging are qualified to interpret coronary artery calcium scoring [32].

### 2. Criteria for interpreting cardiac CT

Physicians should have adequate knowledge and understanding of the anatomy, physiology and pathophysiology of the cardiac systems for cardiac CT interpretation.

#### a) Level 1 competency

This is defined as the minimal introductory training for familiarity with CCTA but is not sufficient for independent interpretation of the CCTA studies [33, 34]. The trainee should have been actively involved in CCTA interpretation under the direction and supervision of a qualified level 2 or 3 mentor.

The trainee should undergo mentored interpretation of at least 25 cases of CCTA with contrast (of which a minimum of 10 cases should be in correlation with conventional coronary angiography). Studies may be taken from an established teaching file or previous CCT cases. Trainees are required to provide proof of training (e.g. verified log book, letter of certification by a qualified level 2 or 3 mentor).

#### b) Level 2 competency

This is defined as the minimum level of training for a doctor to independently perform and interpret CCTA [33, 34]. This is intended for individuals who wish to practise or be actively involved with CCTA. The doctors at this level should have sufficient training to interpret the CT examination accurately and independently.

The trainee should undergo mentored interpretation of at least 75 cases of CCTA, of which the trainee must perform at least 15 cases under supervision of a qualified level 2 or 3 mentor. A minimum of 25 should be correlated with coronary angiography. Studies may be taken from an established teaching file or previous CCTA cases.

The trainee should also undergo training in advanced anatomy, contrast administration, principles of 3-dimensional imaging/post-processing, principles of radiation protection and its hazards to patients and personnel, and appropriate post-procedural patient monitoring.

Trainees are required to provide proof of training (e.g. verified log book, certificate of attendance, letter of certification by a qualified level 2 or 3 mentor)

#### c) Level 3 competency

This represents the highest level of expertise that would enable an individual to serve as a director of a cardiac CT centre [33, 34]. This person would also be directly responsible for quality control and training of radiographers.

This requires a further minimum cumulative training period of 6 months after completion of level 2 training. Trainee should interpret at least 150 cases of CCTA. The cases reflect the broad range of pathology expected in cardiac imaging. The trainee must be involved in performing at least 75 cases and ongoing participation in quality assurance and safety programmes as well as be involved in research activities. Studies may be taken from an established teaching file or previous CCTA cases.

Trainees are required to provide proof of training (e.g. verified log book, certificate of attendance, and letter of certification by a qualified level 3 mentor)

## TRAINING REQUIREMENTS FOR RADIOGRAPHERS

CT radiographers should possess a diploma in radiography, or any equivalent radiography qualifications recognised by the Ministry of Health Malaysia or Society of Radiographers Malaysia.

It is encouraged that the radiographers performing the cardiac CT have advanced certification in at least post-basic CT. The radiographers must also be able to prepare, position, ensure patient safety, monitor the patient, apply the contrast injection and scanning protocol as prescribed by supervising doctors. They should also perform regular quality control testing [32].

If the radiographer does not have an advanced certification in CT, a minimum of 3 months full-time CT training is required under the supervision of a suitably trained CT radiographer and radiologist before being allowed to operate a CT scanner independently. Radiographers are encouraged to keep a log book of the number of CT cases performed.

The radiographer should also have adequate Continuous Professional Development (CPD) on CCTA-related topics (at least attend one conference / workshop / course every 2 years).

CT scanners should not be operated by any person without the above stated qualifications e.g. medical physicists, technicians, research staff, post-doctorate fellows, nurses and any other non-radiological qualified staff.

Intravenous contrast materials can be administered by radiographers and nurses under the direction of supervising doctors, if the practice is in compliance with institutional regulations.

## EXTRACARDIAC FINDINGS OF CORONARY CTA

CT scans performed for cardiac evaluation includes visualisation of extracardiac structures within its scan range. The importance of this is two-fold. Firstly, some of the risk factors for CAD such as smoking, male sex, and age overlap with the risk factors for other chest diseases such as bronchial carcinoma [35].

Secondly, chest pain is not unique to cardiac disease, and may be a result of other chest pathology [36, 37].

With the same raw data, at no additional radiation exposure to the patient, reconstructions of the images into a larger field of view allow visualisation of the lung and chest wall at the level of scan. Dedicated coronary artery CT focused on the heart displays 35.5% of total chest volume, while images reconstructed at maximal field of view visualises 70.3% [35].

Studies have shown that cardiac CT demonstrated a significant number of previously unknown extracardiac findings, some of which had an immediate impact on workup, follow-up or both. The incidence of extracardiac findings ranged from 7.8 to 58.1% [37, 38]. The extracardiac findings that required some form of therapy was 3 to 5% [35, 37]. These included pulmonary embolism, bronchogenic carcinoma, liver tumours and congenital anomalies. Evaluation of extracardiac structures should be performed by a radiologist.

However, cardiac scans have a small field of view restricted to the heart which precludes a complete evaluation of the thorax. Therefore both patients and referring physicians have to understand that the focus of the cardiac CT is for the detection of cardiac diseases.

## APPENDIX

### Pre-Test Probability and 10-year Absolute Risk of Coronary Artery Disease (CAD)

#### Pre-Test Probability

The Pre-test Probability estimates the likelihood of coronary artery disease (defined as presence of angina pectoris, recognised and unrecognised myocardial infarction, unstable angina, and CHD deaths) for any given patient based upon the type of presenting symptoms, age and gender. The risk generally increases with age.

- **High:** Greater than 90% pre-test probability;
- **Intermediate:** Between 10% and 90% pre-test probability;
- **Low:** Between 5% and 10% pre-test probability;
- **Very Low:** Less than 5% pre-test probability.

#### Coronary Artery Disease (CAD) Risk

The risk of developing coronary artery disease during a 10-year period may be calculated from a multifactorial equation recommended by, and summarised in the AHA/ACCF Joint report, ACC/AHA Scientific Statement: Assessment of Cardiovascular Risk by Use of Multiple-Risk-Factor Assessment Equations [21]. The equation takes into account gender, age, total cholesterol, HDL cholesterol, smoking history, presence of left ventricular hypertrophy and diabetes. For example, Low-risk level is defined in the Framingham Report as the risk of coronary heart disease (CHD) at any age for a non-smoker, non-diabetic, with blood pressure less than 120/80 mmHg, total cholesterol of 4.0-5.0 mmol/L, LDL-C 2.5-3.2 mmol/L, and HDL-C greater than or equal to 1.13 mmol/L in men and greater than or equal to 1.39 mmol/L in women.

- **Low Risk:** 10-year absolute risk of less than 10%; generally risk is below age-specific risk level.
- **Medium risk:** 10-year absolute risk 10 to 20%; risk is average or above average of age specific risk level.
- **High Risk:** Presence of Diabetes Mellitus or 10-year absolute risk greater than 20%.

### Notes to Statement

#### Use of words “physician”, “doctor”, “radiologist” and “cardiologist” in this document:

Physician refers to any medically-trained doctor from recognised institutions and registered with the Malaysian Medical Council. A doctor is synonymous with a physician in this document. A radiologist or cardiologist, if specified would refer to a medical doctor/physician who has received the recognised training in the specialty of radiology and cardiology respectively.

**Table 1:** Pre-test Probability of Coronary Artery Disease by Age, Gender and Symptoms (Reproduced from ACC/AHA 2002 Guideline Update for Exercise Testing) [20].

**Age (yrs)**	**Gender**	**Typical/Definite Angina Pectoris**	**Atypical/Probable Angina Pectoris**	**Nonanginal Chest Pain**	**Asymptomatic**
30 - 39	Men	Intermediate	Intermediate	Low	Very low
	Women	Intermediate	Very low	Very low	Very low
40 - 49	Men	High	Intermediate	Intermediate	Low
	Women	Intermediate	Low	Very low	Very low
50 - 59	Men	High	Intermediate	Intermediate	Low
	Women	Intermediate	Intermediate	Low	Very low
60 - 69	Men	High	Intermediate	Intermediate	Low
	Women	High	Intermediate	Intermediate	Low
